# RUVBL1 ubiquitination by DTL promotes RUVBL1/2-β-catenin-mediated transcriptional regulation of NHEJ pathway and enhances radiation resistance in breast cancer

**DOI:** 10.1038/s41419-024-06651-4

**Published:** 2024-04-12

**Authors:** Jie Tian, Mingxin Wen, Peng Gao, Maoxiao Feng, Guangwei Wei

**Affiliations:** 1 Key Laboratory for Experimental Teratology of the Ministry of Education, Department of Cell Biology, School of Basic Medical Sciences, Cheeloo College of Medicine, Shandong University, Jinan, Shandong 250012 China; 2 Key Laboratory for Experimental Teratology of the Ministry of Education, Department of Human Anatomy, School of Basic Medical Sciences, Cheeloo College of Medicine, Shandong University, Jinan, Shandong 250012 China; 3 Key Laboratory for Experimental Teratology of Ministry of Education, Department of Pathology, School of Basic Medical Sciences and Qilu Hospital, Shandong University, Jinan, Shandong 250012 China; 4 Department of Clinical Laboratory, Shandong Provincial Hospital Affiliated to Shandong First Medical University, Jinan, Shandong China

**Keywords:** Breast cancer, Biomarkers

## Abstract

Radiotherapy effectiveness in breast cancer is limited by radioresistance. Nevertheless, the mechanisms behind radioresistance are not yet fully understood. RUVBL1 and RUVBL2, referred to as RUVBL1/2, are crucial AAA+ ATPases that act as co-chaperones and are connected to cancer. Our research revealed that RUVBL1, also known as pontin/TIP49, is excessively expressed in MMTV-PyMT mouse models undergoing radiotherapy, which is considered a murine spontaneous breast-tumor model. Our findings suggest that RUVBL1 enhances DNA damage repair and radioresistance in breast cancer cells both in vitro and in vivo. Mechanistically, we discovered that DTL, also known as CDT2 or DCAF2, which is a substrate adapter protein of CRL4, promotes the ubiquitination of RUVBL1 and facilitates its binding to RUVBL2 and transcription cofactor β-catenin. This interaction, in turn, attenuates its binding to acetyltransferase Tat-interacting protein 60 (TIP60), a comodulator of nuclear receptors. Subsequently, ubiquitinated RUVBL1 promotes the transcriptional regulation of RUVBL1/2-β-catenin on genes associated with the non-homologous end-joining (NHEJ) repair pathway. This process also attenuates TIP60-mediated H4K16 acetylation and the homologous recombination (HR) repair process. Expanding upon the prior study’s discoveries, we exhibited that the ubiquitination of RUVBL1 by DTL advances the interosculation of RUVBL1/2-β-catenin. And, it then regulates the transcription of NHEJ repair pathway protein. Resulting in an elevated resistance of breast cancer cells to radiation therapy. From the aforementioned, it is evident that targeting DTL-RUVBL1/2-β-catenin provides a potential radiosensitization approach when treating breast cancer.

## Background

Breast cancer is the most common type of cancer in women and the second leading cause of cancer-related death in women worldwide [1]. Radiotherapy remains the primary treatment after breast cancer surgery, which not only improve the growth of the primary cancer but also reduces the risk of recurrence and distant metastases [2]. However, a large proportion of breast cancer patients continue to relapse within 10 years after radiotherapy [3], and patients develop resistance to radiotherapy resulting in poor overall survival [4]. Therefore, it is critical to explore the underlying mechanisms that lead to radiation resistance in breast cancer, which will help avoid radiotherapy resistance and improve the survival of breast cancer patients.

The effectiveness of radiation therapy depends on radiation-induced double-stranded DNA breaks (DSBs) in cancer cells, which in turn cause fatal damage to cancer cells. However, when cancer cells develop DSBs, they activate the DNA damage response (DDR) signaling pathway, which consists of DNA damage repair and cell-cycle checkpoints [5, 6]. The main pathways of DSB repair are homologous recombination (HR) and non-homologous terminal splicing (NHEJ) [7]. In human cells, NHEJ repairs almost all DSBs outside the S and G2 phases of the cell cycle. Even within the G2 phase, NHEJ also repairs as much as 80% of ionizing radiation-induced DSBs that are not close to a replication fork [8]. The process of NHEJ repair occurs throughout the cell cycle and connects the broken DNA molecules by splicing, which is prone to chromosome abnormal mutations. In contrast, HR occurs in the S/G2 phase of the cell cycle and requires sister chromatids as templates for repair with high fidelity [9]. In response to ionizing radiation (IR)-induced DSBs, ataxic telangiectasia mutation/Ataxic telangiectasia and Rad3-associated protein (ATM/ATR) kinases are recruited to the DSB lesion site and activated to induce phosphorylation of the histone variant H2AX (γH2AX) or monoubiquitination and polyubiquitination of H2AX/γH2AX. Subsequently, DNA repair-related proteins cluster at damaged DSB sites and complete DNA repair [10, 11]. At the same time, CHK1/2 is activated by ATM/ATR to trigger cell cycle arrest and provide sufficient time for DNA repair [12, 13]. It is well known that dysregulation of key factors involved in DDR can lead to changes in the radiosensitivity of cancer cells [14–16]. Studies have shown that H2AX is necessary for ATM recruitment to DNA damage sites, and that lack of H2AX enhances radiosensitivity [14]. In addition, MYC promotes radioresistance in nasopharyngeal carcinoma cells through transcriptional activation of CHK1 and CHK2 [16]. However, the way DNA repair and cell-cycle control are integrated into DDRs is largely unknown. Therefore, identifying potential key regulatory factors of DDR and understanding the underlying mechanisms involved can provide a theoretical basis for tumor radiation resistance management.

RUVBL1 and RUVBL2 (also known as pontin/TIP49 and reptin/TIP48, respectively) are para-homologous proteins of the AAA+ ATPase family and have homology with bacterial RuvB helicase [17]. As building blocks of diverse macromolecular complexes, the AAA+ ATPases RUVBL1 and RUVBL2 are crucial for many cellular activities including cancer-related processes. Intriguingly, the characteristic oligonucleotide-binding (OB) fold domains (DIIs) of RUVBL1 and RUVBL2 occupy unequal places relative to the compact AAA+ core ring. RUVBL1 and RUVBL2 interact to obtain stability and form annular heterohexers with ATPase activity [18, 19]. RUVBL1/2 is involved in the formation of two protein complexes, the PAQosome [20] and INO80 chromatin remodeling families [21, 22]. In the PAQosome complex, RUVBL1/2 controls the stability of the phosphatidylinositol 3-kinase-associated kinase (PIKK) protein family [23], as well as the biosynthesis of small ribonucleoprotein (snRNP) and kernel ribonucleoprotein (snoRNP) [24–26]. RUVBL1/2 affects nucleosome localization via INO80 [22], histone acetylation via TIP60 [21], and histone composition via SRCAP in INO80 chromatin remodeling complexes [27]. These results suggest that RUVBL1/2 is essential for these complexes. However, under the special environmental pressure of radiotherapy, whether the regulation mode of RUVBL1/2 to form different complexes and play special functions is still unknown.

Cullin‐RING ligases (CRLs) are the largest ubiquitin ligase family in eukaryotes. They are multi‐subunit complexes composed of a cullin scaffold that bridges a ubiquitin-conjugating enzyme (E3) to the substrate. E3 is bound at the cullin C terminus through a RING-finger protein, while the substrate is recognized by a substrate receptor bound through an adapter protein at the cullin N terminus [28]. CRL4-DTL is crucial in preserving the integrity of the genome by ubiquitinating a wide spectrum of factors, directing their proteolysis. CRL4-DTL targets different substrates in a timely manner, thus enabling cell cycle and DNA repair processes to be completed in an ordered way [29]. DTL/CDT2 is an essential component of the early, radiation-induced G2/M checkpoint [30]. DTL mainly mediates the ubiquitination and subsequent degradation of CDT1, Set8, and p21. CDT1 degradation in response to DNA damage is necessary to ensure proper cell-cycle regulation of DNA replication [31, 32]. Moreover, it has been found in the literature that mis‐regulation of DTL has been observed in multiple tumors [33]. In recent years, it has been reported that DTL plays a role in regulating NHEJ in the DNA damage of mammary epithelial cells. It also affects genomic stability and promotes malignant transformation of normal cells. However, whether DTL is involved in the regulation of tumor radiotherapy has not been reported [34].

In this study, we used protein profiling to analyze a spontaneous model of irradiated mouse breast cancer and found that the expression level of RUVBL1 is clearly increased after irradiation. Subsequently, in vitro and in vivo experiments showed that RUVBL1 regulates the resistance of breast cancer cells to radiation therapy. Through mass spectrometry, we also found that DTL modify RUVBL1 through K63 ubiquitination under the condition of radiation treatment, thereby promoting the formation of RUVBL1/2-β-catenin transcription complex. We also revealed that ubiquitination of RUVBL1 by DTL attenuates H4K16 acetylation-mediated HR repair and promotes the expression of NHEJ repair-related proteins. These results suggest that DTL-RUVBL1/2-β-catenin complex has a potential role in breast cancer radiation resistance, and also provides a potential therapeutic target for the clinical treatment of breast cancer radiation resistance.

## Materials and methods

### Radiation model of mouse breast cancer

This study aimed to simulate clinical breast cancer radiation therapy and investigate the molecular mechanism of radiotherapy resistance in breast cancer using a radiation therapy model of adenocarcinoma mice. The model was obtained by subjecting mouse breast cancer spontaneous gene mouse (MMTV-PyMT) to radiation therapy. When the MMTV-PyMT mouse breast cancer tumor grew to an appropriate size (200 mm^3^), they were randomly and blindly divided into control group and radiation group, 7 mice in each group. The radiation group was given five doses of 3 Gy radiation (once every 2 days) [35–37]. Four hours after the last irradiation, the mouse tumors were taken.

### Irradiation of cultured cancer cells

Cells were irradiated with a 2D image-guided Precision X-ray biological irradiator (X-RAD 225 OptiMAX, Institute of Advanced Medical Sciences, Shandong University). The radiation dose rate was 2 Gy/min.

### Sample preparation for LC–MS/MS

Total proteins were extracted from the tumor tissue samples using T-PER buffer (Thermo Fisher Scientific) in the presence of protease inhibitor and phosphatase cocktail (Sigma-Aldrich). The concentration of the soluble proteins was determined by Bicinchoninic Acid (BCA) Protein Assay kit (Pierce, thermo scientific, Germany). Equal amounts of the lysates were desalted followed by reduction using 10 mM dithiothreitol and then alkylation using 50 mM iodoacetamide (IAA) in the dark. For digestion, the proteins were mixed with trypsin (Promega) at a protein:trypsin ratio of 25:1 overnight at 37 °C. Peptides were desalted by ZipTip C18 pipette tips (Millipore), washed with 0.1% trifluoroacetic acid (TFA), and eluted with 50% methanol followed by lyophilizing in a SpeedVac for LC–MS/MS analysis. For pre-fractionation, 50 μg of the peptide mix was re-dissolved in 160 μl of ammonia water (pH = 10) and fractionated by high pressure liquid chromatography (Agilent 1100 system, Agilent Technologies Inc., USA) with a reverse-phase C18 column (250 × 0.1 mm, 3 μm Reprosil). The column was eluted with a 60 min-gradient of acetonitrile from 2 to 50% in ammonia water (pH 10.0). A total of 55 fractions were collected, combined into 10 fractions and lyophilized for LC- MS/MS analysis.

### LC–MS/MS analysis

The MS and MS/MS spectra were acquired by an EASY-nLC 1000 system followed by LTQ-Orbitrap Elite mass spectrometer (Thermo Scientific, San Jose, CA) in data-dependent mode. The spray voltage was 2.1 kV and the capillary temperature 275 °C. MS spectra were acquired in the *m*/*z* range of 350–1800 at a resolution of 60,000 at 400 *m*/*z*. MS/MS fragmentation of the 30 most intense peaks were selected for every full MS scan in the collision-induced dissociation mode. The mass spectrometry proteomics data have been deposited to the ProteomeXchange Consortium (http://proteomecentral.proteomexchange.org) via the iProX partner repository [38, 39] with the dataset identifier PXD047555.

### Immunoprecipitation and mass spectrometry (IP/MS)

Cells were washed with phosphate buffered saline (PBS) and lysed with IP lysis buffer. For the IP assay, protein G/A magnetic beads were washed with IP buffer and crosslinked to the antibodies with rotation for 1 h at room temperature. Then, protein complexes were separated by SDS–PAGE and stained by colloidal coomassie blue. The stained gel slices were excised for the subsequent MS analysis public service platform. IgG was used as a negative control.

### Cell lines and cell cultures

Human breast cancer cell lines (MDA-MB-231, MCF7 and BT549) and HEK293T cells were purchased from American Type Culture Collection (ATCC, Manassas) and HEK293T cell lines were cultured in DMEM, and BT549 cells were maintained with RPMI-1640 medium. The culture medium was supplemented with 10% fetal bovine serum (FBS, BI). These cells were incubated in a humidified incubator containing 5% CO_2_ at 37 °C. The cells were cytogenetic tested and identified before freezing.

### Expression plasmids

The wild-type Flag-DTL plasmids were previously constructed by our lab. The Myc-RUVBL1 and Flag-DTL-HA lentiviral vectors were subsequently constructed. The shRNA sequences targeting DTL were as follows: DTL shRNA #1, GCCTAGTAACAGTAACGAGTA, DTL shRNA #2, CTGGTGAACTTAAACTTGTTA, and DTL shRNA #3, GCTCCCAATATGGAACATGTA. The shRNA sequences targeting β-catenin were as follows: β-catenin shRNA #1, CGCATGGAAGAAATAGTTGAA, β-catenin shRNA #2, GCAACAGTCTTACCTGGA, and β-catenin shRNA #3, GCTTGGAATGAGACTGCTGAT.

### Construction of stable cell line

The Myc-RUVBL1 and a negative control vector co-transfected into HEK293T cells with the lentiviral genomic plasmids. Lentiviral particles were obtained by collecting supernatant for ultracentrifugation concentration and purification of lentiviral particles. Cells were cultured in 6-well plates until 60% confluent and infected with lentivirus particles at a MOI of 50 in the presence of 10 g/ml polybrene for 48 h. Stable cells were maintained in medium containing 600 µg/mL of G418(Beyotime Biotechnology, Shanghai). These cell lines were transfected with DTL or DTL lentiviral short hairpin RNA (shRNA) lentivirus for 48 h. Stable cells were maintained in medium containing 0.5 µg/mL of puromycin (Solarbio, Beijing). The MDA-MB-231-RUVBL1-DTL cell line was transfected with β-catenin lentiviral short hairpin RNA (shRNA) lentivirus for 48 h. Stable cells were maintained in medium containing 25 µg/mL of Blasticidin S (Solarbio, Beijing).

### Cell viability assay

Cells were seeded at a density of 2 × 10^3^ cells per well in 96-well plates in 100 μL of culture medium containing 10% FBS and incubated in a 37 °C, 5% CO_2_ incubator. Cells were treated with IR (0, 1, 2, 4, 6, 8 Gy). After culturing for another 48 h, the MTT reagent (IM0280, Solarbio) was added to each well, and cells were incubated for another 4 h at 37 °C and measured according to the standard procedures.

### Clonogenic cell survival assay

A clonogenic cell survival assay was performed to detect the sensitivity of tumor cells to radiation. Cells of each group were plated in triplicate in a 6-well plate and then irradiated with different X-ray doses (0, 1, 2, 4, 6, 8 Gy). After radiation exposure, the cells were cultured for 10–14 days. When clones were formed (a clone of ≥50 cells were considered a positive clone), the cells were fixed with 4% formaldehyde and incubated with crystal violet solution for 30 min. The clones were counted, and a clone formation curve was drawn.

### Mouse xenograft tumor model

All animal experiments were approved by the Shandong University Animal Care and Use Committee and performed under institute guidelines. Female Balb/c nude mice (4~6 weeks old, 18~20 g) were purchased from Vitong Lihua. Before inoculation with tumor cells, the mice were randomly divided into different groups (5 mice per group) without blinding. The cells of 1 × 10^6^ for experiment and control groups were resuspended with 100 μl PBS and injected subcutaneously into mice. When the tumor size was about 100 mm^3^, a dose of 5 Gy radiation was administered every three days. The length and width of the tumors were measured with vernier calipers every 3 days. Tumor volume calculation formula: *V* = 1/2 × length × width^2^.

### Immunofluorescence

After trypsin digestion and counting, 5 × 10^5^ cells were inoculated into 24-well culture plates for 24 h. After 5 Gy radiation, the cells were incubated for 0, 0.5, 1, 2 h. The cells were cleaned with PBS and fixed at room temperature with 4% paraformaldehyde for 30 min. Cells were infiltrated with 0.5% Triton X-100 at room temperature for 10 min. Subsequently, cells were blocked in 5% goat serum in PBS at room temperature for 1 h and incubated overnight at 4°C with γ-H2AX primary antibody (Cell Signaling Technology, #9718, USA, 1:400). Incubated at room temperature in the dark with the secondary antibody Alexa Fluor 488 conjugated Anti-Rabbit IgG (Invitrogen, USA) for 2 h. Incubate at room temperature for 5 min with DAPI (Beyotime, China). Immunofluorescence staining was observed under laser confocal microscope.

### RNA isolation and qRT-PCR

Total RNA was extracted using TRIzol reagent (Invitrogen) and used for reverse transcription with the PrimeScript RT reagent kit (CWBIO) according to the manufacturer’s instructions. qRT-PCR was performed using the SYBR Green PCR Master Mix (CWBIO) and the ABI PRISM 7900HT Real-time PCR detection system (Eppendorf). GAPDH mRNA levels were used for data normalization. The primers used for quantitative RT-PCR are listed in Supplementary Materials Table 1.

### RNA-sequencing enrichment analysis

RNA was extracted from MDA-MB-231 and MDA-MB-231-RUVBL1 cells, and the sequencing and preliminary data analysis were performed with assistance by LC-bio (Hangzhou, China). An adjusted *p* value of a gene <0.05 and a log2(FC) > 1 were used as the criteria for differentially expressed genes (DEGs) for further data analysis. A gene expression heatmap was generated with an R package (v3.6.3). Gene Ontology (GO) enrichment was performed using the Database for Annotation, Visualization, and Integrated Discovery (DAVID) online tool (http://david.abcc.ncifcrf.gov). NCBI Sequence Read Archive sequencing data were uploaded under accession number PRJNA1044319.

### Statistical analysis

All statistical analyses were performed with Statistical Product and Service Solutions (SPSS) software. Student’s *T*-test and one-way analysis of variance were used to evaluate differences in statistical data. Data are presented as the mean ± SD values, and *p* < 0.05 was considered to indicate a significant difference. More information of the materials and methods is in the Supplementary Materials.

## Result

### RUVBL1 plays an important role in breast cancer radiotherapy

**Fig. 1:**
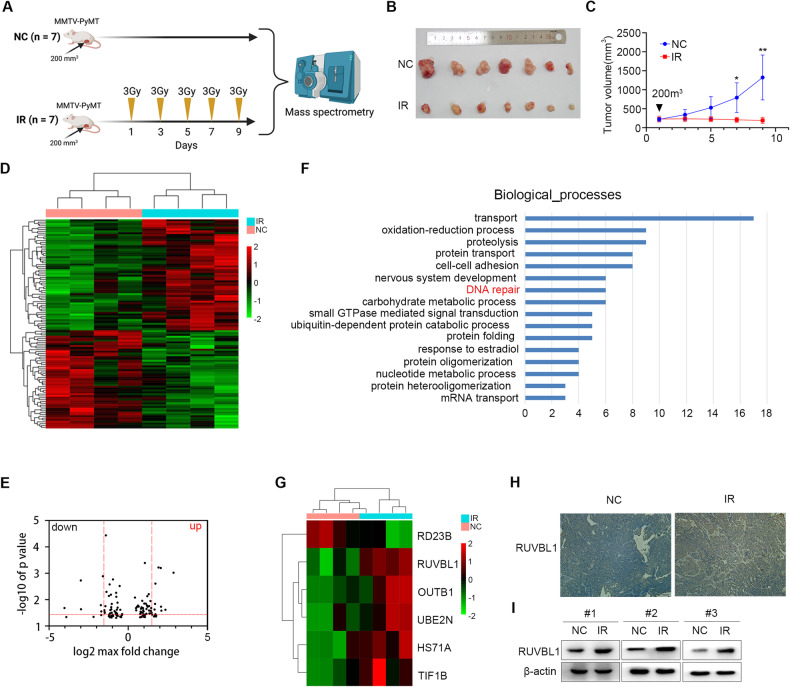
RUVBL1 expression is comparatively high in the radiation breast-tumor tissue. **A** FVB-TgN (MMTV-PyMT) transgenic female mice were irradiated when their tumors reached 200 mm^3^. Every 2 days, 3 Gy each time. **B** The irradiated mice were dissected and the tumors were photographed and compared at the end of the experiment. **C** Tumor growth was monitored every 2 days for 5 times, and the volume was recorded till the experiment ended (*n* = 7 mice). **D** Heatmaps showing the 348 differentially expressed proteins in irradiated and non-irradiated tumor tissue. **E** Volcano plot comparing control and irradiated tumor tissue. **F** Enrichment of differentially expressed proteins in signaling pathways is portrayed as a bar chart. Y-axis represents pathways, and the X-axis represents rich factor. **G** Heatmap of DNA repair pathway. **H** Immunohistochemical (IHC) for RUVBL1 in tumor tissues (×10). Scale bars: 100 μm. **I** Western blot for RUVBL1 and β-actin in tumor tissues. Data presented as mean ± SD, *p < 0.05, statistical differences were assessed using two-tailed unpaired Student’s *t*-test in (**C**). (**p < 0.01).

To study the mechanism of radiation resistance in breast cancer, MMTV-PyMT (mouse mammary tumor virus-polyoma virus middle T antigen, MMTV-PyMT) [40, 41] mice were used to construct a breast cancer radiotherapy model. When the breast cancer tumors in mice grew to a certain size (200 mm^3^), the treatment group was given 5 consecutive intervals of radiation therapy (3 Gy) (Fig. 1A). As expected, the growth of irradiated tumors was significantly controlled relative to controls (Fig. 1B, C).

To explore the differences in proteomics in breast cancer after radiation therapy, we collected mouse breast cancer tissues for mass spectrometry analysis. After mass spectrometry analysis, we identified a total of 2130 proteins, of which 148 proteins were significantly increased in the radiation-treated group, while 200 proteins were significantly decreased in the radiation-treated group (Fig. 1D, E). Further functional enrichment analysis of these differentially expressed proteins showed that these proteins were mainly involved in biological processes such as protein transfer, intercellular adhesion, DNA repair, carbohydrate metabolism, and ubiquitination-dependent protein degradation (Fig. 1F). Since DNA repair is closely related to tumor radiation resistance, we first focused on these differentially expressed proteins in DNA repair process. Among these differential proteins related to DNA repair, we found that RUVBL1 was consistently high expression level in different tumor tissues treated with radiation (Fig. 1G). Subsequent studies in radiation-treated or untreated mouse mammary tumor tissues also confirmed that RUVBL1 was highly expressed in the radiation-treated group (Fig. 1H, I). Simultaneously, we also verified the expression of other proteins in the pathway, which was consistent with the omics results (Supplementary Fig. 1). However, the difference in protein expression was not significant. Therefore, we selected RUVBL1 for further investigation. These results suggest that radiation therapy induces the expression of RUVBL1, and also suggest that RUVBL1 may play a regulatory role in breast cancer radiation resistance.

### High expression of RUVBL1 enhances radiation tolerance in breast cancer

**Fig. 2:**
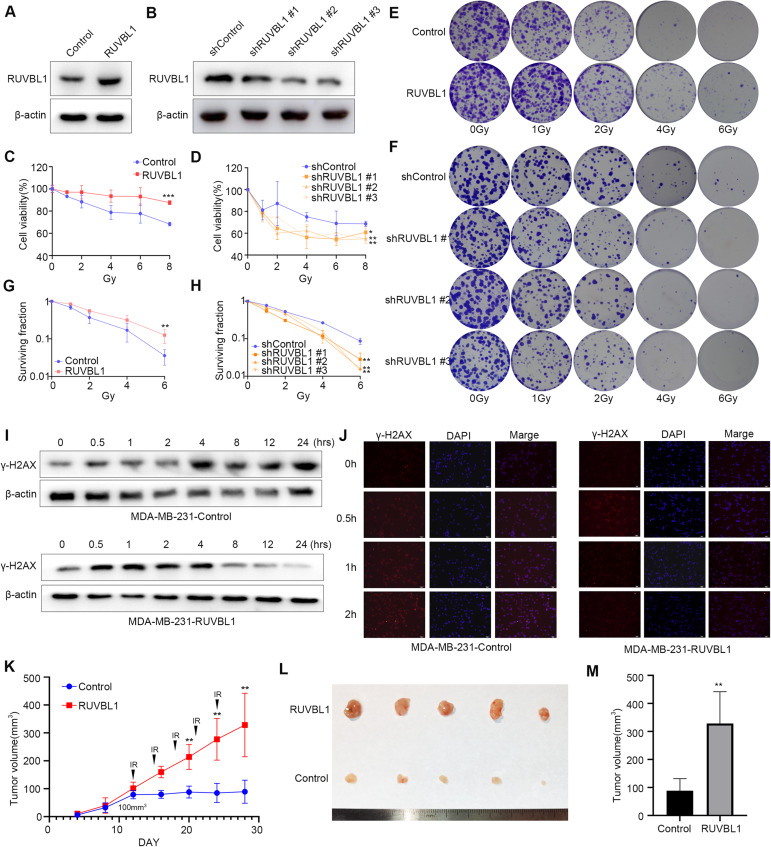
RUVBL1 regulates radiation resistance of breast cancer cells. **A** Western blot for RUVBL1 and β-actin in MDA-MB-231 RUVBL1 cell line. **B** Western blot for RUVBL1 and β-actin in MDA-MB-231 shRUVBL1 cell line. **C**, **D** The stable cell lines underwent a series of radiation treatments at 0, 1, 2, 4, 6 and 8 Gy, followed by an MTT test (*n* = 3). **E**–**H** The same cell lines were subjected to radiation at 0, 1, 2, 4, and 6 Gy, prior to being cloned and evaluated for survival curve (*n* = 3). **I** Western blot for γ-H2AX and β-actin in MDA-MB-231 RUVBL1 cells with radiation (5 Gy). **J** Immunofluorescence for γ-H2AX in MDA-MB-231 RUVBL1 cells with radiation (5 Gy) (×10). Scale bars: 100 μm. **K** 2 × 10^5^ control or RUVBL1 MDA-MB-231 cells were subcutaneously injected into nude mice (*n* = 5). Since the 12th day, each group of nude mice were treated with radiation (Every 3 days, 3 Gy each time). Tumor growth curves were shown. Tumors (**L**) and tumor size (**M**) of mice were shown. Data presented as mean ± SD, *p < 0.05, statistical differences were assessed using two-tailed unpaired Student’s *t*-test (**C**, **E**, **F**, **H**, **K**, **M**). (*p < 0.05, **p < 0.01, ***p < 0.001).

Previous studies have shown that RUVBL1 regulates tumor drug resistance and is involved in regulating the HR repair pathway in DNA damage [42, 43], but its function in breast cancer radiation resistance is poorly understood. To further clarify the role of RUVBL1 in breast cancer radiation resistance, we constructed cell lines stably overexpressing or knocking down RUVBL1 in breast cancer cells (MAD-MB-231, MCF7 and BT549) (Fig. 2A, B and Supplementary Fig. 2A, B). These cell lines were subsequently irradiated at different doses, and the results showed that overexpression of RUVBL1 significantly increased cell viability and proliferation (Fig. 2C, E, G and Supplementary Fig. 2C–H). Conversely, knocking down RUVBL1 significantly reduced cell viability and proliferation (Fig. 2D, F, H). Furthermore, we also detected the level of DNA damage in breast cancer cells overexpressing RUVBL1 after radiation treatment, and the results showed that overexpressing RUVBL1 significantly reduced the expression level of γ-H2AX protein in breast cancer cells (Fig. 2I, J). Finally, breast cancer cells overexpressing RUVBL1 were inoculated into nude mice, and irradiated continuously after 12 days of tumor growth (Fig. 2K and Supplementary Fig. 2I). The results showed that overexpression of RUVBL1 significantly increased tumor growth and weight (Fig. 2L, M and Supplementary Fig. 2J, K). These results suggest that RUVBL1 regulates the resistance of breast cancer cells to radiation therapy.

To further investigate whether RUVBL1 play a role in the resistance of breast cancer cells to radiotherapy, we have constructed MDA-MB-231 radioresistant cell lines (MDA-MB-231 RR). Our analysis revealed that MDA-MB-231 RR displayed greater levels of radioresistant compared to normal MDA-MB-231 cells (supplementary Fig. 3A). Furthermore, it was found that RUVBL1 exhibited high expression levels in MDA-MB-231-RR cells, which resulted in an increased level of radiation tolerance (Supplementary Fig. 3B–D). This indicates that RUVBL1 plays a significant role in the radiation tolerance of breast cancer.

### DTL ubiquitinates RUVBL1 via ubiquitin K63

**Fig. 3:**
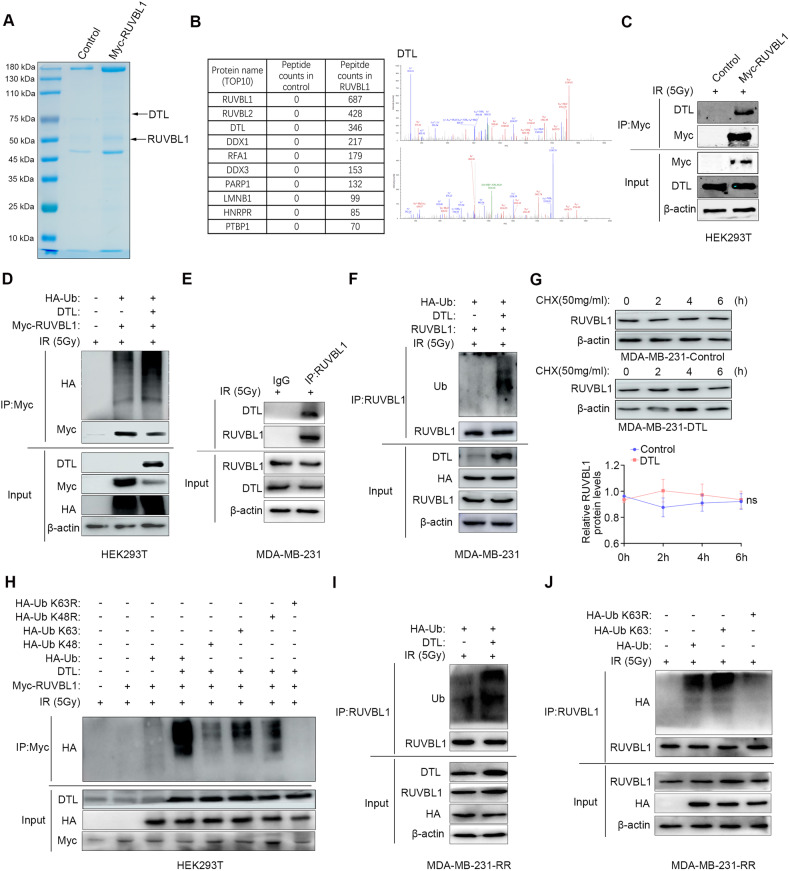
RUVBL1 enhances DNA damage repair and is ubiquitinated by DTL. **A**, **B** HEK 293T cells transfected with control vector or Myc-RUVBL1 were immunoprecipitated with Myc antibody and subjected to mass spectrometry analysis. DTL was identified as a novel binding partner for RUVBL1. **C** HEK293T cells transfected with Myc-RUVBL1 were harvested for immunoprecipitation with Myc antibody, followed by western blot. **D** HEK293T cells transfected with Myc-RUVBL1, DTL or HA-Ub were immunoprecipitated with Myc antibody and western blot analysis. **E** MDA-MB-231-RUVBL1 cells were harvested for immunoprecipitation with RUVBL1 antibody, followed by western blot. **F** MDA-MB-231-RUVBL1 cells transfected with DTL and HA-Ub were immunoprecipitated with RUVBL1 antibody and western blot analysis. **G** RUVBL1 half-life is unchanged in MDA-MB -231-RUVBL1 compared with control cells. Cells were switched to fresh medium (10% FBS) containing cycloheximide (CHX) for indicated time periods (0, 2, 4, 6 h) and harvested for western blot. The band density was quantified (*n* = 3). **H** HEK 293T cells transfected with Myc-RUVBL1, DTL, HA-Ub, HA-Ub K48, HA-Ub K63, HA-Ub K48R or HA-Ub K63R were immunoprecipitated with Myc antibody and analyzed by western blot. **I** MDA-MB-231-RUVBL1-RR cells transfected with DTL and HA-Ub were immunoprecipitated with RUVBL1 antibody and western blot analysis. **J** MDA-MB-231-RR cells transfected with HA-Ub, HA-Ub K63, or HA-Ub K63R were immunoprecipitated with RUVBL1 antibody and analyzed by western blot. Data presented as mean ± SD, *p < 0.05, statistical differences were assessed using two-tailed unpaired Student’s *t*-test (**G**). ns non-significant.

To further clarify the mechanism by which RUVBL1 promotes resistance to radiation therapy in breast cancer cells, we used CoIP and mass spectrometry to search for proteins to which RUVBL1 potentially binds (Fig. 3A). Mass spectrometry and co-immunoprecipitation confirmed that RUVBL1 could bind to DTL (Fig. 3B, C). Our previous studies have shown that DTL regulates the accumulation of DNA damage in normal cells by affecting the stability of DNA-PKcs protein [44]. Therefore, we speculated that DTL may be involved in the regulation of RUVBL1 on the radiation resistance of breast cancer cells. Studies have shown that DTL, as a substrate recognition receptor for CUL4A E3 ubiquitin-conjugating enzyme, affects the stability and function of the target proteins by specifically binding to the ubiquitination modification of the target proteins [44–47]. Therefore, we detected the ubiquitination level of RUVBL1 and the results showed that DTL-ubiquitinated RUVBL1 in HEK293T cells (Fig. 3D). We also verified the results in breast cancer cells, MDA-MB-231 cells (Fig. 3E, F), and the results were consistent with those in HEK293T cells. RUVBL1 binds to DTL and DTL promotes RUVBL1 ubiquitination (Fig. 3E, F). Subsequent protein half-life results showed that overexpression of DTL did not affect the stability of RUVBL1 protein (Fig. 3G). These results suggest that DTL ubiquitination of RUVBL1 may be a monobitination modification of the ubiquitin (Ub) K63 site. The results of further ubiquitination experiments showed that the ubiquitization modification of RUVBL1 by DTL was Ub K63 mode (Fig. 3H). These results suggest that DTL may affect the function of RUVBL1 and thus regulate the radiation resistance of breast cancer cells.

We examined the effect of DTL on RUVBL1 ubiquitination after radiation in MDA-MB-231-RR cells and found that DTL promoted RUVBL1 ubiquitination (Fig. 3I). Furthermore, we analyzed the interaction between RUVBL1 and ubiquitin mutated at different sites. We conducted immunoprecipitation experiments in MDA-MB-231-RR cells to verify the interaction between RUVBL1 and ubiquitin K63. We observed a robust binding between these proteins when only K63 sites were present (Fig. 3J). This was consistent with the results in MDA-MB-231 cells. The results indicate that DTL plays a crucial role in the radiation tolerance of breast cancer cells by ubiquitinating RUVBL1 at the K63 site.

### RUVBL1 regulates breast cancer radiation resistance in a DTL-dependent manner

**Fig. 4:**
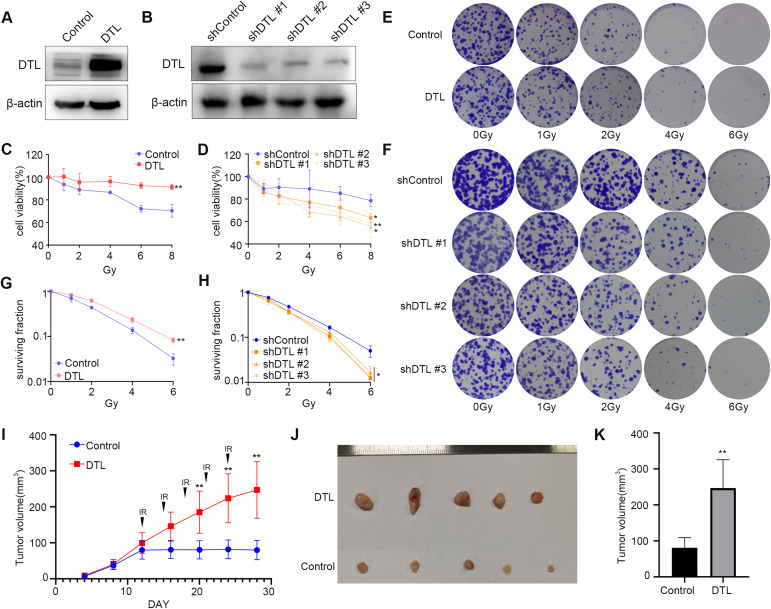
DTL regulates radiation resistance of breast cancer cells. **A** Western blot for DTL and β-actin in MDA-MB-231 DTL stably expressed cells. **B** Western blot for DTL and β-actin in MDA-MB -231 shDTL stably expressed cells. **C**, **D** The stable cell lines underwent a series of radiation treatments at 0, 1, 2, 4, 6, and 8 Gy, followed by an MTT test (*n* = 3). **E**–**H** The stable cell lines were subjected to radiation at 0, 1, 2, 4, and 6 Gy, prior to being cloned and evaluated for survival curve (*n* = 3). **I** 2 × 10^5^ control or RUVBL1 MDA-MB-231 cells were subcutaneously injected into nude mice (*n* = 5). Since the 12th day, each group of nude mice were treated with radiation (Every 3 days, 3 Gy each time). Tumor growth curves were shown. Tumors (**J**) and tumor size (**K**) of mice were shown. Data presented as mean ± SD, *p < 0.05, statistical differences were assessed using two-tailed unpaired Student’s *t*-test (**C**, **D**, **G**, **H**, **I**, **K**) (*p < 0.05, **p < 0.01).

Considering that RUVBL1 is ubiquitinated by DTL in breast cancer cells, we assumed that DTL might exert a radioresistance effect in breast cancer. To test this hypothesis, we constructed cell lines overexpressing or knocking down DTL in breast cancer cells (MDA-MB-231, MCF7 and BT549) (Fig. 4A, B and Supplementary Fig. 4A, B). These breast cancer cells were subsequently irradiated with different radiation doses, and overexpression of DTL significantly increased cell viability and proliferation (Fig. 4C, E, G and Supplementary Fig. 4C–H). In contrast, knockdown of DTL significantly reduced cell viability and proliferation (Fig. 4D, F, H). Finally, breast cancer cells overexpressing DTL were inoculated into nude mice, and irradiated continuously after 12 days of tumor growth (Fig. 4J and Supplementary Fig. 4J). The results showed that overexpression of DTL significantly increased tumor growth and weight (Fig. 4I–K and Supplementary Fig. 4I–K). These results suggest that DTL also has the function of regulating breast cancer radiation resistance, similar to the function of RUVBL1.

**Fig. 5:**
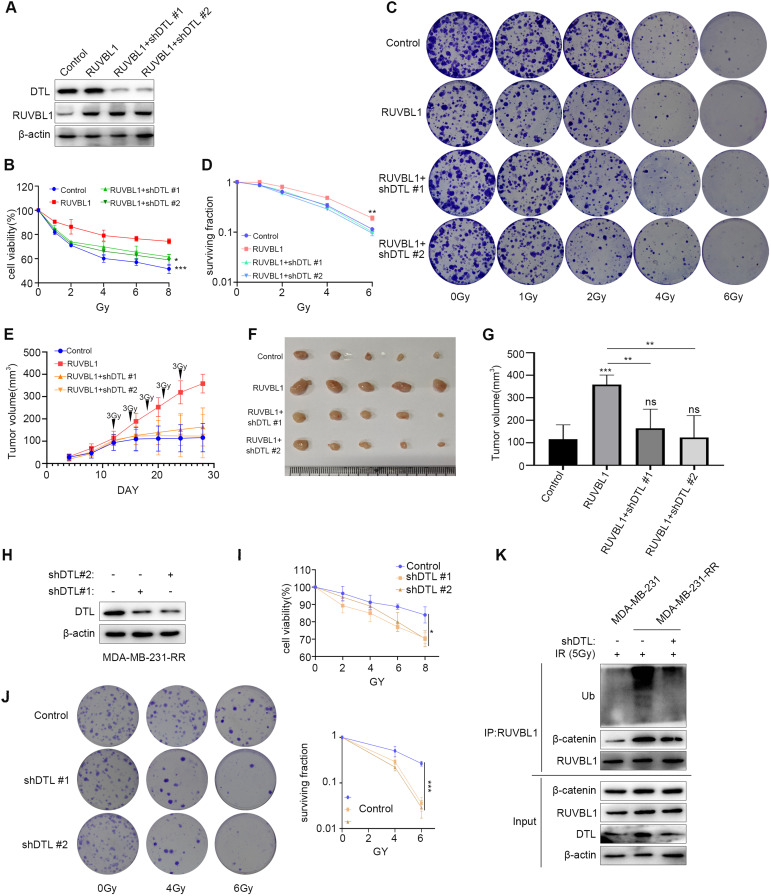
RUVBL1 regulates radiation resistance of breast cancer cells by DTL. **A** Western blot for RUVBL1, DTL and β-actin in MDA-MB-231 control, RUVBL1, RUVBL1-shDTL#1 and RUVBL1-shDTL#2 stably expressed cells. **B**–**D** MDA-MB-231 control, RUVBL1, RUVBL1-shDTL#1 and RUVBL1-shDTL #2 cells were treated with radiation (0, 1, 2, 4, 6, 8 Gy), followed by MTT assay and clonogenic survival assay (*n* = 3). **E** 2 ×10^5^ MDA-MB-231 control, RUVBL1, RUVBL1-shDTL #1 and RUVBL1-shDTL #2 cells were subcutaneously injected into nude mice (*n* = 5). Since the twelfth day, each group of nude mice were treated with radiation (Every 3 days, 3 Gy each time). Tumor growth curves were shown. Tumors (**F**) and tumor size (**G**) of mice were shown. **H** Western blot assay was used to detect the expression of DTL in MDA-MB-231-RR. **I** The stable cell lines underwent a series of radiation treatments at 0, 4, and 6 Gy, followed by an MTT test (*n* = 3). **J** The MDA-MB-231-RR Control and shDTL cells were subjected to radiation at 0, 4, and 6 Gy, prior to being cloned and evaluated for survival curve (*n* = 3). **K** MDA-MB-231-RR cells transfected with shDTL were immunoprecipitated with RUVBL1 antibody and western blot analysis. Data presented as mean ± SD, *p < 0.05, statistical differences were assessed using two-tailed unpaired Student’s *t*-test (**B**, **D**, **E**, **G**, **I**, **J**). (*p < 0.05, **p < 0.01, ***p < 0.001).

To further understand the role of RUVBL1 and DTL in the regulation of breast cancer radiation resistance, we knocked down DTL in RUVBL1-overexpressing MDA-MB-231 cells to analyze the resistance of breast cancer to radiation therapy (Fig. 5A). Subsequently, these cells were irradiated with different doses of radiation, and knockdown of DTL clearly reversed the effect of RUVBL1 on enhancing the radiation resistance of breast cancer cells (Fig. 5B–D). In vivo tumorigenesis of RUVBL1-overexpressing and DTL-knockdown cells followed by radiation therapy was also showed similar results (Fig. 5E–G). These results suggest that DTL is involved in RUVBL1-mediated radiation resistance of breast cancer.

We examined the change of radiation resistance after DTL knockdown in MDA-MB-231-RR cell line (Fig. 5H), and found that the radiation sensitivity of MDA-MB-231-RR cells increased (Fig. 5I, J). Moreover, we found that RUVBL1 ubiquitination levels decreased after DTL knockdown (Fig. 5K). This suggests that DTL-RUVBL1 plays an important role in MDA-MB-231-RR radiation tolerance.

### DTL ubiquitinates RUVBL1 to attenuates H4K16 acetylation

**Fig. 6:**
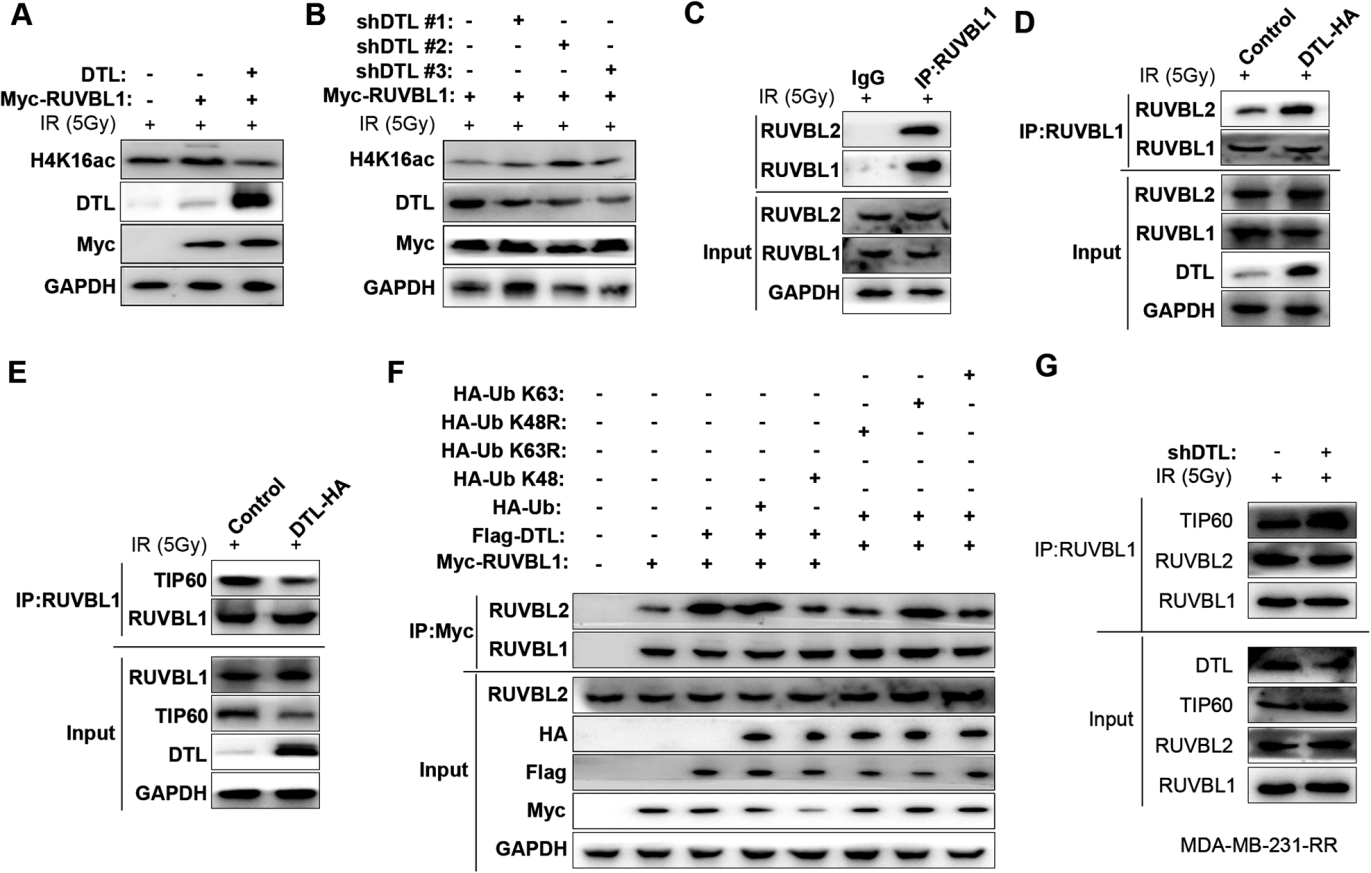
DTL-ubiquitinated-RUVBL1 attenuates H4K16 acetylation-mediated HR repair pathway. **A** The changes of H4K16ac level after high expression of RUVBL1 and DTL were detected by western blot. **B** The changes of H4K16Ac level after high expression of RUVBL1 and knockdown of DTL were detected by western blot. **C** The analysis of the interaction between RUVBL1 and RUVBL2 was carried out in MDA-MB-231-RUVBL1 cells using immunoprecipitation (IP) after 4 h of IR, and it was compared with IgG. **D**–**F** HEK293T cells were transfected with the indicated plasmids prior to treatment with IR for 4 h before collection. The lysates were incubated with Myc antibody overnight and then subjected to western blot. **G** MDA-MB-231-RR cells were transfected with the shDTL plasmids prior to treatment with IR for 4 h before collection. The lysates were incubated with RUVBL1 antibody overnight and then subjected to western blot.

Previous studies have shown that methylated RUVBL1 binds to TIP60 to acetylate H4K16, thereby promoting the HR repair process of DNA double-strand breaks [43]. Although our subsequent experimental results showed that overexpression of RUVBL1 could significantly promote the level of H4K16ac, overexpression of DTL significantly reduced the acetylation modification of H4K16 by RUVBL1 (Fig. 6A). Consistently, DTL knockout significantly promoted the acetylation of H4K16 by RUVBL1 (Fig. 6B). Subsequently, it was found that DTL significantly promoted the interaction between RUVBL1 and RUVBL2, and inhibited the interaction between RUVBL1 and TIP60 (Fig. 6C, D). Further experiments also confirmed that the K63 ubiquitination modification of RUVBL1 by DTL promoted the interaction between RUVBL1 and RUVBL2 (Fig. 6E, F). Subsequently, we observed that in MDA-MB-231-RR cells, when DTL levels were low, the binding of RUVBL1 to RUVBL2 was reduced. However, the combination of RUVBL1 and TIP60 was increased (Fig. 6G). The modification of RUVBL1 by DTL through ubiquitination increased its interaction with RUVBL2, but inhibited the interaction of RUVBL1/2 with TIP60. These results suggest that DTL-RUVBL1 does not promote the HR pathway through TIP60-H4K16ac.

### Ubiquitination of RUVBL1 by DTL promotes the formation of RUVBL1/2-β-catenin complexes

**Fig. 7:**
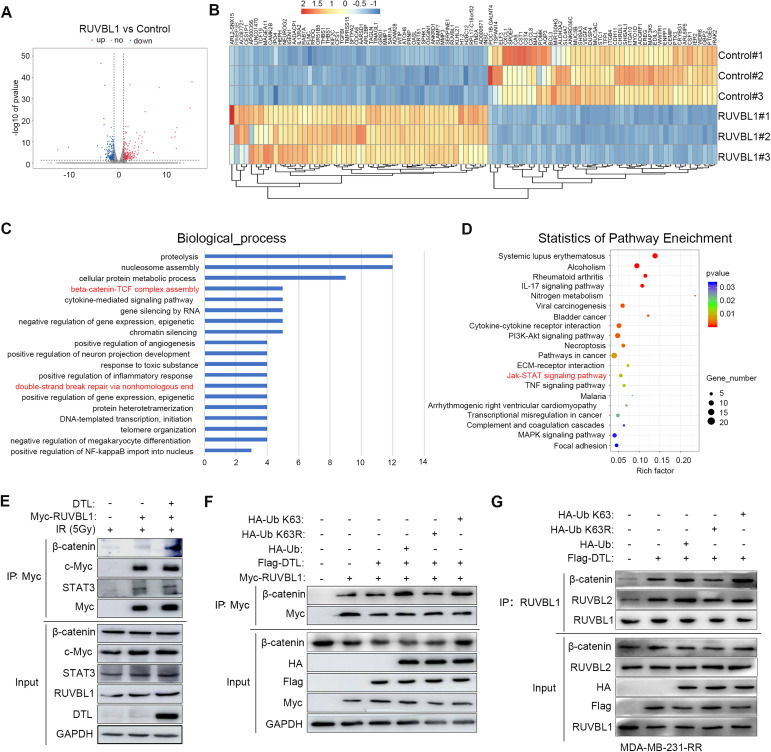
DTL promotes the binding of RUVBL1 to transcription cofactor β-catenin. **A** Volcano plot comparing control and cells high expression of RUVBL1 after IR 4 h. **B** Heat map from RNA-sequencing analysis. **C** Enrichment of differentially expressed genes in GO signaling pathways is illustrated in a bar graph. Y-axis represents pathways, and the X-axis represents rich factor. **D** Enrichment of differentially expressed genes in signaling pathways is illustrated in an advanced bubble chart. Size and color of the bubble are representation of the amount of differentially expressed genes enriched in pathways and their enrichment significance, respectively. **E** Immunoprecipitation (IP) analysis of the interaction between RUVBL1 and β-catenin, c-Myc, STAT3 in HEK293T cells after IR 4 h. **F** HEK293T cells transfected with Myc-RUVBL1, DTL, HA-Ub, HA-Ub K63 or HA-Ub K63R were immunoprecipitated with Myc antibody and western blot analysis. **G** MDA-MB-231-RR cells transfected with Flag-DTL, HA-Ub, HA-Ub K63 or HA-Ub K63R were immunoprecipitated with RUVBL1 antibody and western blot analysis.

In order to further explore how ubiquitination of RUVBL1 by DTL regulates the radiation resistance of breast cancer cells, we then performed transcriptomic analysis (RNA-seq) on radiation-irradiated breast cancer cells stably overexpressing RUVBL1. We found significant changes in the expression of 547 genes in cells with high ruvbl1 expression compared to control cells (*P* value < 0.05 and foldchange absolute value ≥ 2) (Fig. 7A, B). Functional enrichment analysis of differentially expressed genes revealed that β-catenin complex assembly, non-homologous end repair of double-strand breaks (NHEJ) and Jak-STAT signaling pathways were enriched, and these signaling pathways were associated with cellular radiation resistance (Fig. 7C, D). Subsequently, through analysis of transcription factors and auxiliary factor related to radiation and DNA damage in the literature, β-catenin, c-Myc, and STAT3 were found. These transcription -related factors are involved in radiation resistance and are also related to RUVBL1. And these three factors are also reflected in our RNA-Seq [2, 48–53]. Further co-immunoprecipitation results showed that RUVBL1 could interact with β-catenin, c-Myc and STAT3 respectively (Fig. 7E). However, overexpression of DTL only significantly increased the interaction of RUVBL1 with β-catenin (Fig. 7E). Subsequent ubiquitination experiments further verified that RUVBL1 modified by K63 ubiquitination of DTL increased the interaction with β-catenin (Fig. 7F). This was also verified in MDA-MB-231-RR cells. When Ub-K63 is present, RUVBL1 binds strongly to RUVBL2 and β-catenin (Fig. 7G). These results suggest that DTL-ubiquitinated-RUVBL1 promotes the interaction of RUVBL1 with β-catenin.

### Ubiquitination of RUVBL1 by DTL promotes expression of NHEJ repair molecules through β-catenin-mediated transcriptional regulation

**Fig. 8:**
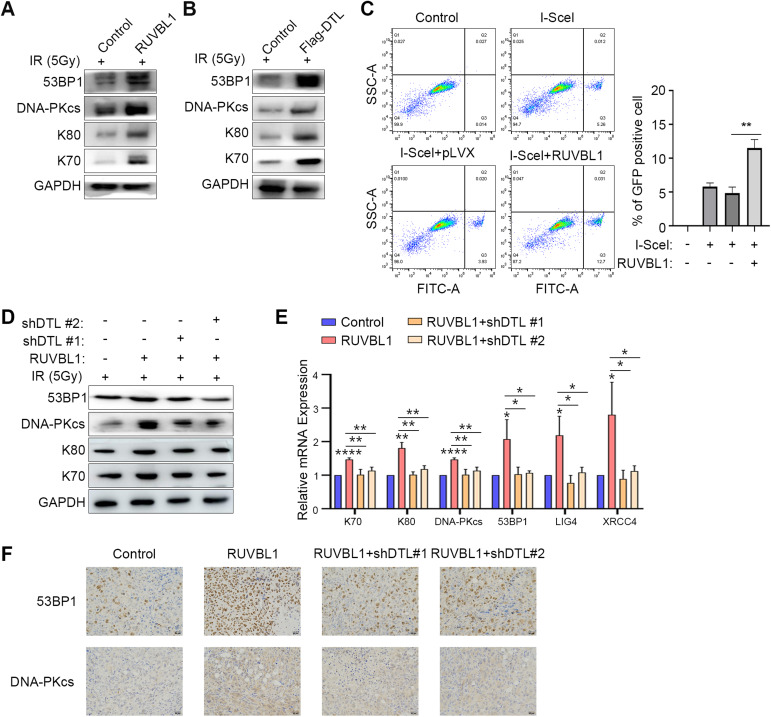
RUVBL1 affects the expression of NHEJ pathway genes and is regulated by DTL. **A** The expressions of NHEJ pathway gene in MDA-MB-231 control and RUVBL1 cell s and corresponding normal cells were determined by western blot, radiate in 5 Gy for 4 h. **B** The expressions of NHEJ pathway genes in MDA-MB-231 control and DTL cells and corresponding normal cells were determined by western blot, radiate in 5 Gy for 4 h. **C** DSBs were induced through the I-SceI method, using the NHEJ reporter gene system. Subsequently, flow cytometry was utilized to analyze the impact of RUVBL1 on NHEJ efficiency. **D** The expressions of NHEJ pathway genes in MDA-MB-231 control, RUVBL1 and RUVBL1-shDTL cells and corresponding normal cells were determined by western blot, radiate in 5 Gy for 4 h. **E** qRT-PCR detected the mRNA level of the NHEJ pathway genes in MDA-MB-231 control, RUVBL1 and RUVBL1-shDTL cell s, radiate in 5 Gy for 4 h (*n* = 3). **F** The expression of DNA-PKcs and 53BP1 in the nude tumor tissues was detected by IHC (×20). Scale bars: 50 μm. Data presented as mean ± SD, statistical differences were assessed using two-tailed unpaired Student’s *t*-test (**C**, **E**). (*p < 0.05, **p < 0.01, ***p < 0.001, ****p < 0.0001).

Our previous results found that DTL-ubiquitinated-RUVBL1 attenuates the HR repair pathway mediated by H4K16 acetylation, and RNA-seq analysis suggested that RUVBL1 regulated the NHEJ repair pathway. Therefore, we speculated that DTL-RUVBL1 may enhance the radiation resistance of breast cancer cells by regulating NHEJ repair. Subsequently, it was discovered that the overexpression of RUVBL1/DTL greatly increased the expression of NHEJ repair pathway molecules, namely K70, K80, DNA-PKcs, 53BP1, LIG4, and XRCC4, within breast cancer cells (Fig. 8A, B). The outcomes of the reporter gene also suggested that the activation of the NHEJ repair pathway was initiated by RUVBL1(Fig. 8C). Conversely, it was noted that the overexpression of RUVBL1/DTL significantly reduced the expression of HR repair-related molecules in the Supplementary Fig. 5A, B. Compared to normal breast cancer cells, radiotolerant cells showed an increase in the expression of the NHEJ pathway protein, while the change in the HR pathway protein was not significant (Supplementary Fig. 5C, D). This finding is consistent with the high expression of RUVBL1/DTL, indicating the activation of the NHEJ pathway after radiation exposure. DTL knockdown significantly reversed RUVBL1-increased expression of NHEJ repair molecules (Fig. 8D–F), while HR repair molecules did the opposite (Supplementary Fig. 5E, F). These results indicated that RUVBL1-DTL increased the radiation resistance of breast cancer cells by enhancing the NHEJ repair pathway in breast cancer cells.

**Fig. 9:**
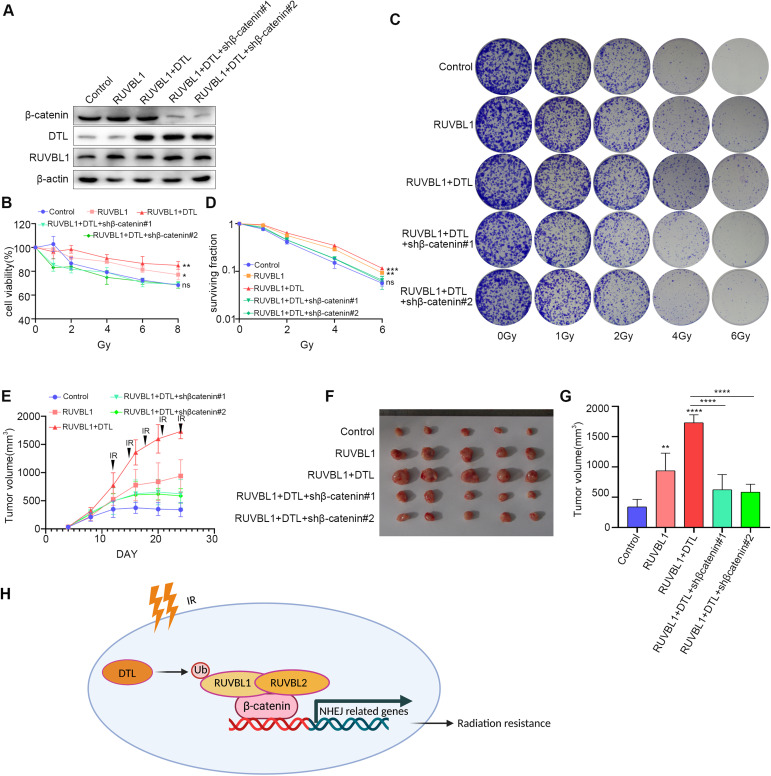
DTL-RUVBL1-β-catenin regulates radiation resistance of breast cancer cells. **A** Western blot for RUVBL1, DTL, β-catenin and β-actin in MDA-MB-231 control, RUVBL1, RUVBL1-DTL, RUVBL1-DTL-shβ-catenin#1 and RUVBL1-DTL-shβ-catenin#2 stably expressed cells (HA-DTL; Myc-RUVBL1). **B**–**D** The above cell lines were treated with radiation (0, 1, 2, 4, 6, 8 Gy), followed by MTT assay and clonogenic survival assay (*n* = 3). **E** 2 × 10^5^ the above cells were subcutaneously injected into nude mice (*n* = 5). Since the twelfth day, each group of nude mice were treated with radiation (Every 3 days, 3 Gy each time). Tumor growth curves were shown. Tumors (**F**) and tumor size (**G**) of mice were shown. **H** Schematic diagram. RUVBL1 ubiquitination by DTL promotes RUVBL1/2-β-catenin-mediated transcriptional regulation of NHEJ pathway and enhances radiation resistance in breast cancer. Data presented as mean ± SD, statistical differences were assessed using two-tailed unpaired Student’s *t*-test (**B**, **D**, **E**, **G**). *p < 0.05, **p < 0.01, ***p < 0.001, ****p < 0.0001, ns non-significant.

Since we have found that DTL ubiquitination promotes the interaction between RUVBL1/2 and β-catenin, we then tried to verify if β-catenin mediates the regulation of RUVBL1/DTL on the expression of NHEJ repair pathway molecules. Knockdown of β-catenin in breast cancer cells overexpressing RUVBL1 and DTL significantly reduced the expression level of NHEJ molecules (Supplementary Fig. 6A, B, D). Moreover, we observed an increase in the NHEJ repair effect when DTL expression was high, while knockdown of β-Catenin reduced the NHEJ repair benefits of RUVBL1 and DTL (Supplementary Fig. 6C). This finding supports our conclusion that DTL-RUVBL1-β-catenin enhances breast cancer’s radiation tolerance through the promotion of the NHEJ repair pathway. Knockdown of β-catenin significantly reduced RUVBL1-DTL-mediated radiation resistance of breast cancer in vitro and in vivo (Fig. 9A–G). These results suggested that DTL-ubiquitinated-RUVBL1 enhances the radiation resistance of breast cancer cells by regulating the expression of NHEJ repair molecules through β-catenin transcription (Fig. 9H).

## Discussion

Radiotherapy, as an option for locoregional cancer treatment, is increasingly utilized in the postoperative management of breast cancer [54], particularly with the recognition of the effectiveness for the treatment after breast-conserving surgery and in the early-stage of breast cancer. After breast-conserving surgery, radiotherapy to the conserved breast halves the rate of the disease recurrence and reduces the breast cancer death rate by about one sixth. These proportional benefits vary little between different groups of women. By contrast, the absolute benefits from radiotherapy vary substantially according to the characteristics of the patients and the benefits can be predicted at the time when treatment decisions need to be made [55]. However, radioresistance leads to relapse and makes breast cancer refractory. Radioresistance of breast cancer cells can lead to poor prognosis, including radiotherapy failure and the accumulation of toxic effects associated with large doses of ionizing radiation. The radiation response of cancer cells is complex and mainly determined by their intrinsic repair ability, which is controlled at a genetic level. In this study, we found that ubiquitination of RUVBL1 by DTL enhances the radiation resistance of breast cancer cells. Furthermore, we found that DTL-ubiquitinated-RUVBL1 promotes the formation of RUVBL1/2-β-catenin complex, which in turn promoted the expression of NHEJ repair molecules at transcriptional level, which in turn increases the NHEJ pathway activity and finally causes the radiation resistance of breast cancer tumor cells (Fig. 9H).

RUVBL1 and RUVBL2 (collectively RUVBL1/2) are essential AAA+ ATPases that function as co-chaperones and have been implicated in cancer. RUVBL1 and RUVBL2 are essential proteins that interact with and stabilize a diverse array of multiprotein complexes. RUVBL1/2 ATPase activity is increased in patients with non-small cell lung cancer, and the activity drives PAQosome maturation, DNA replication and radioresistance in lung cancer [56]^.^ However, the mechanism of how RUVBL1/2 regulates tumor radiation resistance is still unclear. In this study, we elucidated that ubiquitination of RUVBL1 by DTL promotes the formation of RUVBL1/2-β-catenin complex, which in turn promotes the NHEJ repair pathway of breast cancer cells to resist radiation therapy.

DTL plays an essential role in many biological processes, including DNA damage response, regulation of cell cycle, protein monoubiquitination and polyubiquitination [57]. The CRL4Cdt2 ubiquitin ligase is responsible for the regulation of multiple substrates during S phase and after the induction of DNA damage, which are important for cell-cycle control and the DNA damage response10. Many of these factors are important for ensuring once-per-cell-cycle DNA replication (e.g., Cdt1, Cdc6, Set8), timely cell-cycle progression (e.g., p21, E2F1, CHK1), accurate repair of the genome following DNA damage (polymerase η and δ, XPG, TDG, SDE2, FBH1) and in linking the cell cycle to DNA damage responses (Cdt1, Set8, p21) [29]. It also mono‐ubiquitinates PCNA in S phase, to facilitate translesion synthesis (TLS) [58]. Importantly, following DNA damage, DTL-PANC inhibits the HR pathway and promotes the TLS pathway by ubiquitinating FBH1 [59]. This suggests that DTL is not restricted to a single pathway in cell injury and can facilitate cell repair through multiple pathways. DTL can degrade DNA-PKcs in epithelial cells, increase genomic instability, and promote normal cell carcinogenesis [44]. However, the role of DTL in breast cancer cell radiotherapy tolerance has not been clearly established. Additionally, NHEJ repairs up to 80% of ionizing radiation-induced DSBs that are not close to a replication fork [8]. The study reveals that DTL-RUVBL1-β-catenin promotes the NHEJ pathway and affects the radiation tolerance of breast cancer cells. It also suggests that DTL not only affects the HR pathway but also promotes faster DNA repair in the NHEJ pathway when radiation-induced DSB occurs, thus promoting breast cancer radiotherapy tolerance.Protein ubiquitination is closely associated with cancers by regulating both tumor-suppressing and tumor-promoting pathways [60, 61]. Studies have shown that DTL expression is up-regulated in breast cancer tissues, but how it plays a role in radiotherapy for breast cancer has not been reported. In this study, we found that RUVBL1 interacts with DTL in radiation-irradiated breast cancer cells. Subsequent experiments confirmed that DTL ubiquitinates RUVBL1 through ubiquitin K63, which is generally believed to contribute to protein function regulation. Subsequently, we confirmed that DTL plays a regulatory role in the radiation resistance of breast cancer cells, and mediates the effect of RUVBL1 on the radiation resistance of breast cancer cells.

We found that RUVBL1 increased in combination with RUVBL2 after ubiquitination by DTL. RUVBL1 and its binding partner RUVBL2 are present in a number of separate high molecular weight nuclear complexes, containing TIP60, SRCAP, or INO80, that regulate a variety of cellular processes, including the DSB response [62–68]. Methylated RUVBL1 is critically required for the acetyltransferase activity of TIP60, promoting histone H4K16 acetylation, which facilities 53BP1 displacement from DSBs, promoting HR pathway activity. However, we found that DTL-ubiquitinated-RUVBL1 attenuates the interaction between RUVBL1 and TIP60, thereby reducing the acetylation level of H4K16 in breast cancer cells. Instead, ubiquinated RUVBL1 increases its binding with RUVBL2 and β-catenin, which in turn enhances the expression of NHEJ pathway core elements. These results suggested that RUVBL1 is modified by DTL to inhibit the HR repair pathway and may induce radiation resistance through the NHEJ repair pathway.

It is reported that LIG4, a DNA ligase in DNA double-strand break repair, is a direct target of β-catenin. Wnt signaling enhances NHEJ repair in CRC, which is mediated by LIG4 transactivation by β-catenin [69]. In this study, DTL-RUVBL1 not only regulates the NHEJ pathway, but also promotes the binding of RUVBL1 and β-catenin. Therefore, we speculated whether DTL-RUVBL1 affects β-catenin activity on gene expression in NHEJ pathway. Our results showed that DTL-RUVBL1-β-catenin increases the expression of NHEJ repair pathway molecules at transcriptional level, thereby enhancing the radiation resistance of breast cancer cells.

Although radiation therapy is an increasingly common adjuvant therapy for breast cancer, radiation resistance of cancer cells remains a major limitation to be overcome. Gaining insight into the molecular mechanisms of radiation resistance will facilitate the development of radiosensitizers for cancer therapy. Our results suggested that DTL ubiquitination of RUVBL1 promotes the formation of RUVBL1/2-β-catenin complex, thereby regulating the transcription of NHEJ repair pathway molecules and enhancing the resistance of breast cancer cells to radiation therapy. Through the research on the mechanism of DTL-RUVBL1/2-β-catenin in the radiation resistance of breast cancer, it is expected to systematically reveal the mechanism of radiation resistance of breast cancer cells, and also provide new ideas for the treatment of breast cancer radiation resistance.

### Supplementary information

Supplementary materials

Original Data File

## Data Availability

The mass spectrometry proteomics datasets generated during the current study are available in the ProteomeXchange Consortium repository (http://proteomecentral.proteomexchange.org) via the iProX partner repository [38, 39] with the dataset identifier PXD047555. The RNA-sequencing datasets generated during the current study are available in the NCBI repository, https://www.ncbi.nlm.nih.gov/bioproject/PRJNA1044319/.
