# Supplementary material for: RUVBL1 ubiquitination by DTL promotes RUVBL1/2-β-catenin-mediated transcriptional regulation of NHEJ pathway and enhances radiation resistance in breast cancer
Source: Cell Death Dis. 2024 Apr 12;15(4):259. doi: 10.1038/s41419-024-06651-4 (PMC11015013; doi:10.1038/s41419-024-06651-4)
Supplement: Supplementary file 1 — Supplementary materials [file 41419_2024_6651_MOESM1_ESM.docx]

**Supplementary information for**

**RUVBL1 ubiquitination by DTL promotes RUVBL1/2-β-catenin-mediated transcriptional regulation of NHEJ pathway and enhances radiation resistance in breast cancer**

Jie Tian^1^, Mingxin Wen^2^, Peng Gao^3^, Maoxiao Feng^4, *^, Guangwei Wei^1, *^

1 Key Laboratory for Experimental Teratology of the Ministry of Education, Department of Cell Biology, School of Basic Medical Sciences, Cheeloo College of Medicine, Shandong University, Jinan, Shandong250012, China.

2 Key Laboratory for Experimental Teratology of the Ministry of Education, Department of Human Anatomy, School of Basic Medical Sciences, Cheeloo College of Medicine, Shandong University, Jinan, Shandong 250012, China

3 Key Laboratory for Experimental Teratology of Ministry of Education, Department of Pathology, School of Basic Medical Sciences and Qilu Hospital, Shandong University, Jinan, Shandong 250012, China.

4 Department of Clinical Laboratory, Shandong Provincial Hospital Affiliated to Shandong First Medical University, Jinan, Shandong, China.

* Corresponding Authors: Maoxiao Feng: School of Basic Medical Sciences, Cheeloo College of Medicine, Shandong University, 44 Wenhua West Road, Jinan, Shandong, China. Tel: +86-531-88382312, E-mail: fengmaoxiao@sdu.edu.cn; Guangwei Wei: School of Basic Medical Sciences, Cheeloo College of Medicine, Shandong University, 44 Wenhua West Road, Jinan, Shandong, China. Tel: +86-531-88382312, E-mail: gwwei@email.sdu.edu.cn

**Supplementary table**

**Supplementary Table 1** Sequences of qRT-PCR primers used in this study

| **Genes** | **Forward primer (5’-3’)** | **Reverse primer (5’-3’)** |
| --- | --- | --- |
| Ku70  Ku80  DNA-PKcs  53BP1  LIG4  XRCC4  RAD51  MRE11  RPA32  BRAC1  Polδ  GAPDH | CATGGCAACTCCAGAGCAG  AGAAGAAGGCCAGCTTTGAG  CCGGACGGACCTACTACGACT  GGCTACGCATTTCTCCTTACC  AGCCTGACCTGGAGAACAGA  CATTGTTGTCAGGAGCAGGA  CTCAGCCTCCCGAGTAGTTG  GCCTTCCCGAAATGTCACTA  CCAGTGGGTTGACACAGATG  CTCAAGGAACCAGGGATGAA  AGAAAGCCATGCTAAAGGACAG  CGACCACTTTGTCAAGCTCA | GCTCCTTAAACTCATCCACC  AGCTGTGACAGAACTTCCAG  AGAACGACCTGGGCATCCT  AAGCTGGGATTCTGTATACTGC  CATGCAGGCTTGACAACATC  TCTGCAGGTGCTCATTTTTG  CATCACTGCCAGAGAGACCA  TTCAAAATCAACCCCTTTCG  TGATAGGTGCTCTCCCTGCT  GCTGTAATGAGCTGGCATGA  CATGTGAAGATGTGACTGCTCA  GGGTCTTACTCCTTGGAGGC |

**Supplementary Table 2** Antibodies used in this study

| **Antibodies** | **Catalog#** | **Source** |
| --- | --- | --- |
| anti-β-actin  anti-RUVBL1  anti-CDT2（DTL）  anti-Myc-Tag  anti-HA  anti-GAPDH  anti-H4K16ac  anti-RUVBL2(Reptin)  anti-TIP60(KAT5)  anti-Flag  anti-β-catenin  anti-c-MYC  anti-STAT3  anti-53BP1  anti-DNA-PKcs  anti-K70  anti-K80  anti-RAD51  anti-MRE11  anti-BRCA1  anti-γ-H2AX  anti-γ-H2AX  anti-OTUB1  anti-UBE2N  anti-HS71A  anti-RAD23B  anti-TIF1B | TA-09  PA5-29278  NOVUS  Cell Signaling  Sigma-Aldrich  abcam  Zenbio  Zenbio  Zenbio  abcam  Cell Signaling  Cell Signaling  Zenbio  Zenbio  abcam  abcam  abcam  abcam  Zenbio  Zenbio  abcam  Milipore  abcam  abcam  Solarbio  abcam  Solarbio | ZSGB-BIO  Invitrogen  NB100-40840  #2276S  H3663  ab8245  380899  R381952  221268  F3165-5MG  # 8480S  # 18583S  R22785  R381816  ab32566  ab92450  ab80952  ab133534  382493  342823  ab26350  05-636  ab220959  ab105164  K1015213P  ab223776  K106665P |

**Supplementary figures**


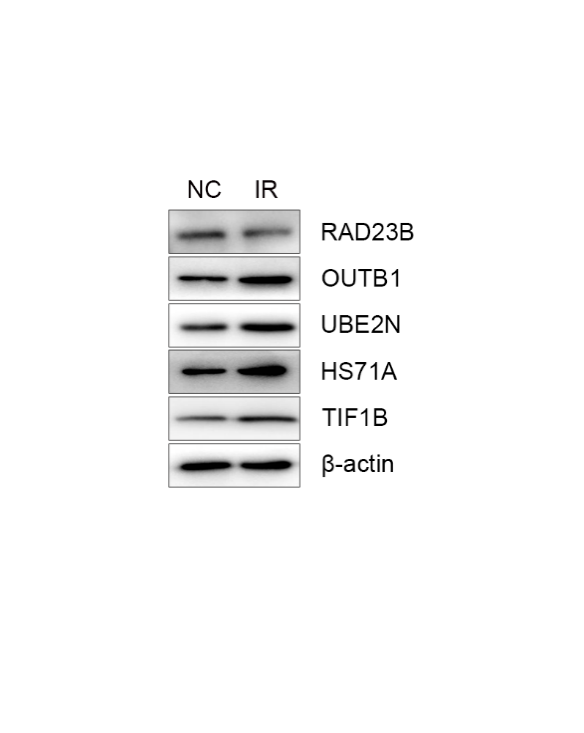


**Supplementary Figure 1. DNA repair pathway protein validation in mass spectrometry results.** Western blot assay was used to detect the expression of RAD23B, OUTB1, TIF1B, UBE2N in mouse breast cancer radiotherapy model.


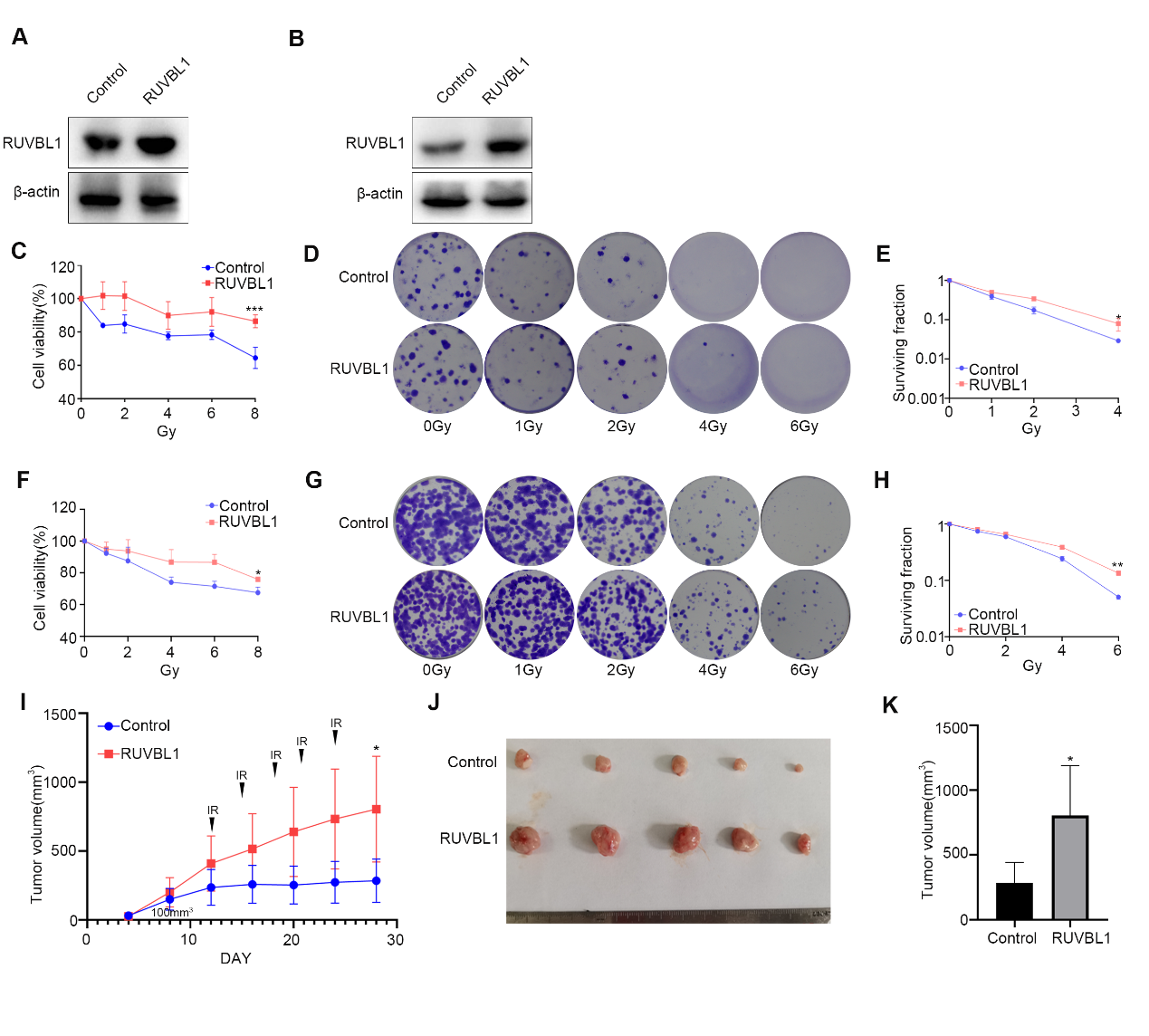


**Supplementary Figure 2. RUVBL1 regulates radiation resistance of breast cancer cells.** (A-B) Western blot for RUVBL1 in BT-549 and MCF7 RUVBL1 stably expressed cell lines. (C-E) BT 549 Control and RUVBL1 high expression cell lines were treated with radiation (0Gy, 1Gy, 2Gy, 4Gy, 6Gy, 8Gy), followed by MTT assay and clonogenic survival assay (n = 3). (F-H) MCF7 Control and RUVBL1 high expression cell lines were treated with radiation (0Gy, 1Gy, 2Gy, 4Gy, 6Gy, 8Gy), followed by MTT assay and clonogenic survival assay (n = 3). (I) 2 x105 control or RUVBL1 MCF7 cells were subcutaneously injected into nude mice (n = 5). Since the twelfth day, each group of nude mice were treated with radiation (Every 3 days, 3 Gy each time). Tumor growth curves were shown. (J-K) Tumors (J) and tumor size (K) of mice were shown. Data presented as mean ± SD, * p < 0.05, statistical differences were assessed using two-tailed unpaired Student’s t test (C, E, F, H, I, K). (*p < 0.05, **p < 0.01, ***p < 0.001)


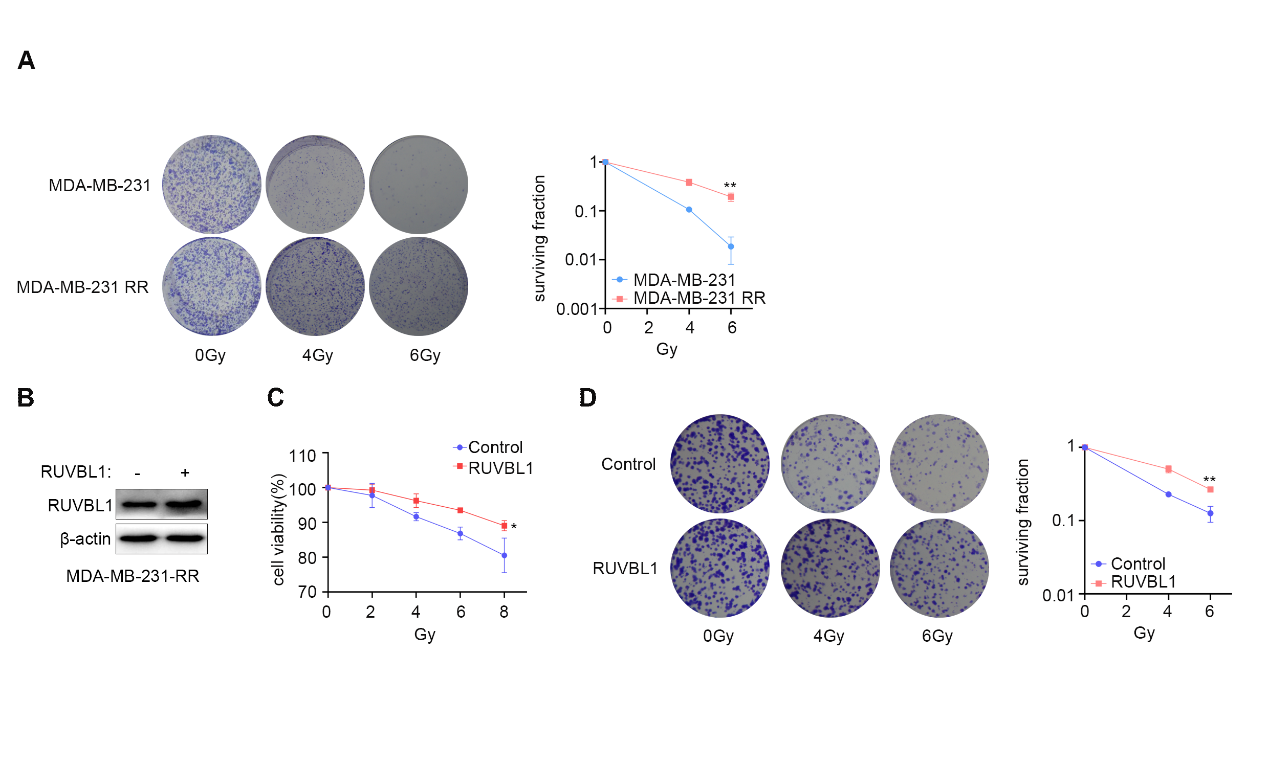


**Supplementary Figure 3. RUVBL1 increases the radiation tolerance of MDA-MB-231.** (A). The MDA-MB-231 and MDA-MB-231 RR cells were subjected to radiation at 0 Gy, 4 Gy, and 6 Gy, prior to being cloned and evaluated for survival curve (n = 3). (B). Western blot assay was used to detect the expression of RUVBL1 in MDA-MB-231-RR. (C). The stable cell lines underwent a series of radiation treatments at 0 Gy, 4 Gy, and 6 Gy, followed by an MTT test (n = 3). (D). The MDA-MB-231-RR Control and RUVBL1 cells were subjected to radiation at 0 Gy, 4 Gy, and 6 Gy, prior to being cloned and evaluated for survival curve (n = 3). Data presented as mean ± SD, * p < 0.05, statistical differences were assessed using two-tailed unpaired Student’s t test (A, C, D). (*p < 0.05, **p < 0.01)


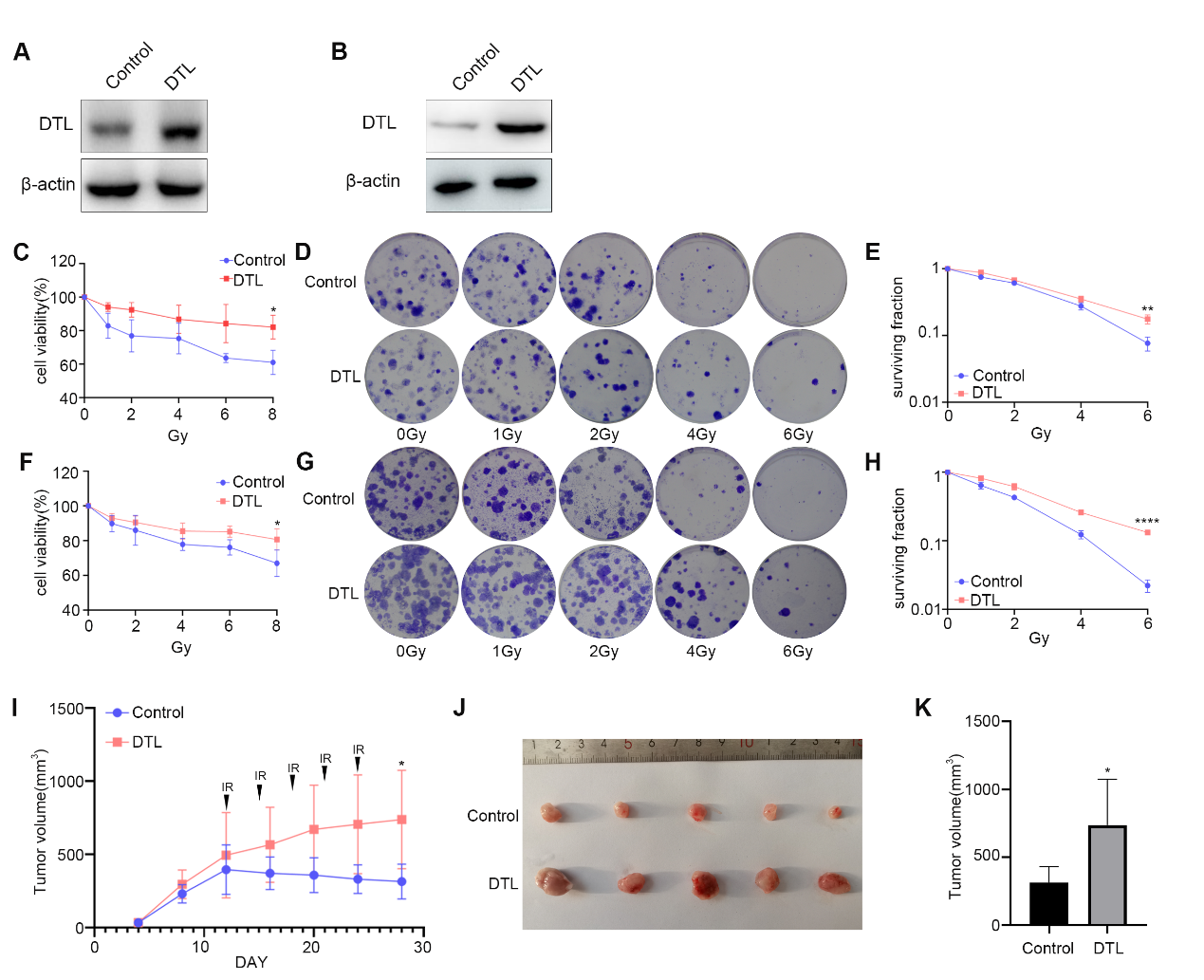


**Supplementary Figure 4. DTL regulates radiation resistance of breast cancer cells.** (A-B) WB for DTL in BT-549 and MCF7 DTL stably expressed cell lines. (C-E) BT-549 Control and DTL high expression cell lines were treated with radiation (0Gy, 1Gy, 2Gy, 4Gy, 6Gy, 8Gy), followed by MTT assay and clonogenic survival assay (n = 3). (F-H) MCF7 Control and DTL high expression cell lines were treated with radiation (0Gy, 1Gy, 2Gy, 4Gy, 6Gy, 8Gy), followed by MTT assay and clonogenic survival assay (n = 3). (I) 2 x105 control or RUVBL1 MDA-MB-231 cells were subcutaneously injected into nude mice (n = 5). Since the twelfth day, each group of nude mice were treated with radiation (Every 3 days, 3 Gy each time). Tumor growth curves were shown. (J-K) Tumors (J) and tumor size (K) of mice were shown. Data presented as mean ± SD, * p < 0.05, statistical differences were assessed using two-tailed unpaired Student’s t test (C, E, F, H, I, K). (*p < 0.05, **p < 0.01, ****p < 0.0001)


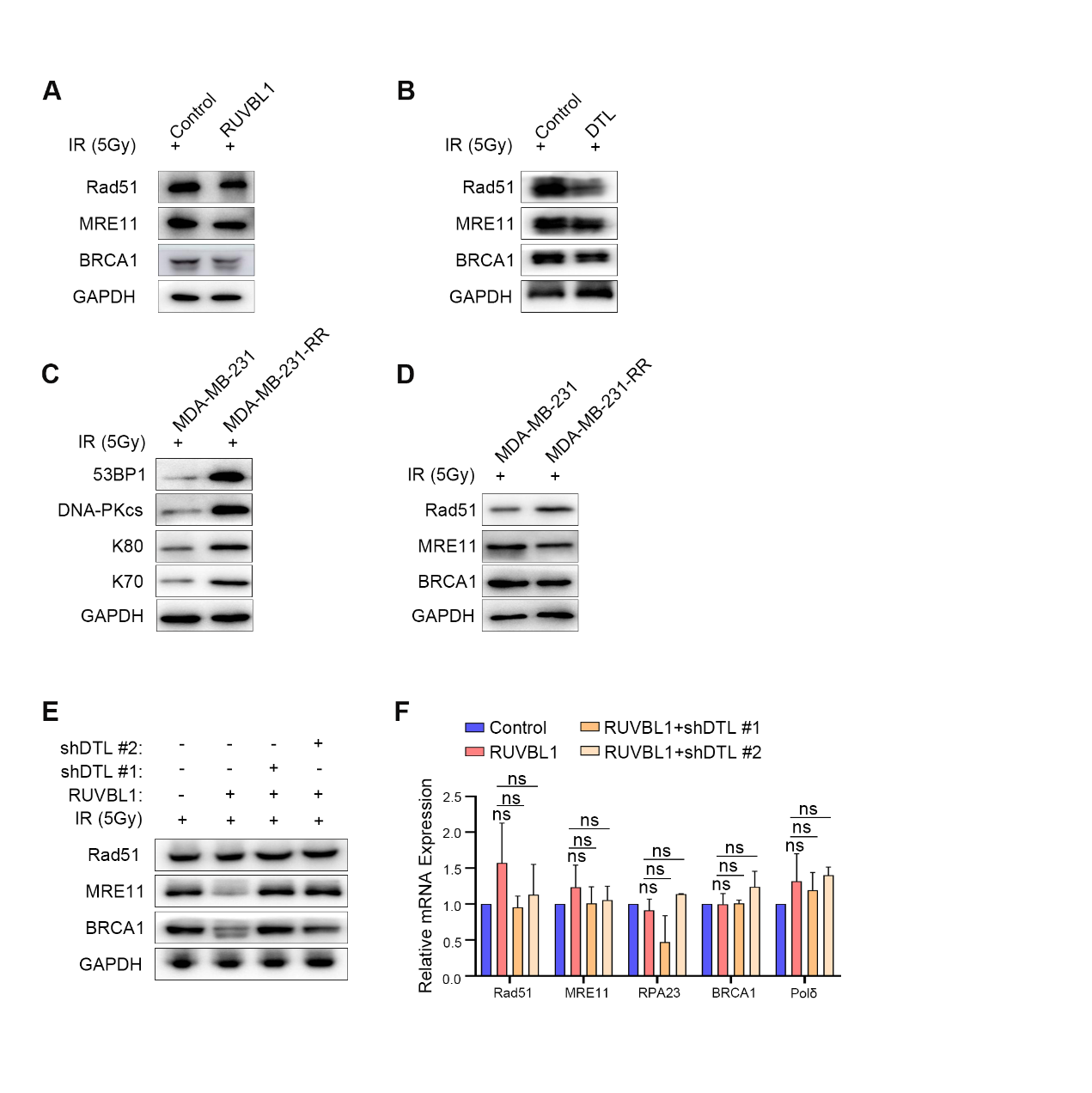


**Supplementary Figure 5. MDA-MB-231-RR activates the NHEJ path and DTL-RUVBL1 does not affect the expression of HR path genes.** (A) The expressions of HR pathway gene in the MDA-MB-231 Control and RUVBL1 cell lines and corresponding normal cells were determined by western blot, radiate in 5Gy for 4 hours. (B) The expressions of HR pathway gene in the MDA-MB-231 Control and DTL cell lines and corresponding normal cells were determined by western blot, radiate in 5Gy for 4 hours. (C) Western blot assay was used to detect the expression of NHEJ pathway-associated proteins in MDA-MB-231-RR. (D) Western blot assay was used to detect the expression of HR pathway marker proteins in MDA-MB-231-RR. (E) The expressions of HR pathway proteins in MDA-MB-231 Control, RUVBL1, RUVBL1-shDTL#1 and RUVBL1-shDTL#2 cell lines and corresponding normal cells were determined by western blot, radiate in 5Gy for 4 hours. (F) Q-PCR detect the mRNA expression level of the HR pathway gene in MDA-MB-231-Control, RUVBL1, RUVBL1-shDTL#1 and RUVBL1-shDTL#2 cell lines, radiate in 5Gy for 4 hours (n = 3). Data presented as mean ± SD, * p < 0.05, statistical differences were assessed using two-tailed unpaired Student’s t test (F). (Non-significant=ns)


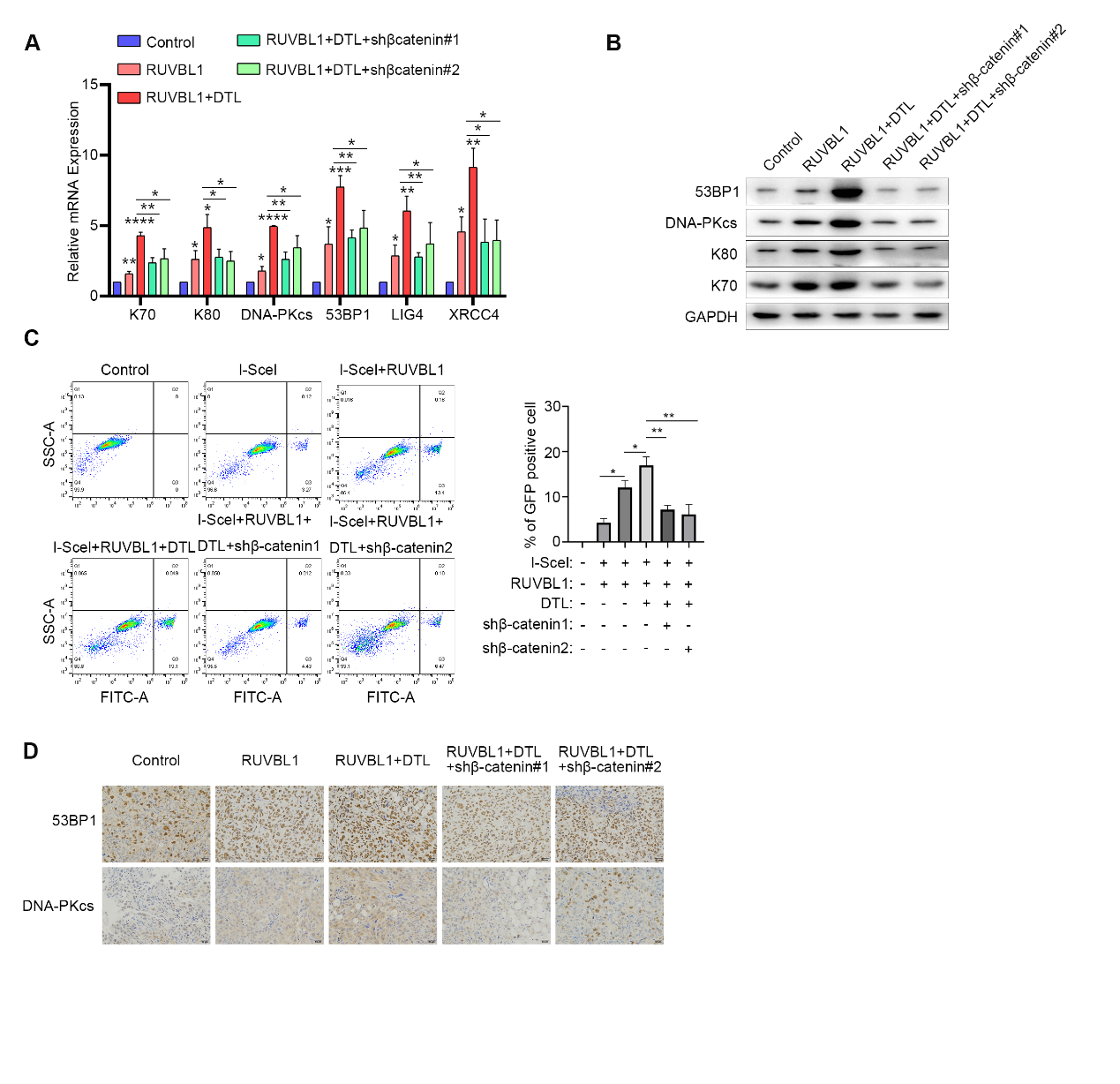


**Supplementary Figure 6. DTL-RUVBL1 affects the expression of NHEJ pathway genes and is regulated by β-catenin.** (A) Q-PCR detect the mRNA expression level of the NHEJ pathway gene in MDA-MB-231 Control, RUVBL1, RUVBL1+DTL, RUVBL1+DTL+shβ-catenin #1 and RUVBL1+DTL+shβ-catenin #2 cell lines, radiate in 5Gy for 4 hours (n = 3). (B) The expressions of NHEJ pathway proteins in the above cell lines and corresponding normal cells were determined by western blot, radiate in 5Gy for 4 hours. (C) DSBs were induced through the I-SceI method using an NHEJ reporter gene system. The impact of RUVBL1, DTL and β-catenin on NHEJ efficiency was analysed by flow cytometry. (n = 3) (D) The expression of DNA-PKcs and 53BP1 in the nude tumor shown was detected by IHC (×20). Scale bars: 50 μm. Data presented as mean ± SD, * p < 0.05, statistical differences were assessed using two-tailed unpaired Student’s t test (A, C). (*p < 0.05, **p < 0.01, ***p < 0.001, ****p < 0.0001)
